# Metabopolis: scalable network layout for biological pathway diagrams in urban map style

**DOI:** 10.1186/s12859-019-2779-4

**Published:** 2019-04-15

**Authors:** Hsiang-Yun Wu, Martin Nöllenburg, Filipa L. Sousa, Ivan Viola

**Affiliations:** 1 Research Division of Computer Graphics, Institute of Visual Computing and Human- Centered Technology, TU Wien, Vienna, Austria; 2 Algorithms and Complexity Group, Institute of Logic and Computation, TU Wien, Vienna, Austria; 3 Archaea Biology and Ecogenomics Division, Department of Ecogenomics and Systems Biology, University of Vienna, Vienna, Austria; 4 Computer Science, Computer, Electrical and Mathematical Science and Engineering, King Abdullah University of Science and Technology (KAUST), Thuwal, Saudi Arabia

**Keywords:** Biological pathways, Graph drawing, Map metaphor, Orthogonal layout, Floor planning, Edge routing

## Abstract

**Background:**

Biological pathways represent chains of molecular interactions in biological systems that jointly form complex dynamic networks. The network structure changes from the significance of biological experiments and layout algorithms often sacrifice low-level details to maintain high-level information, which complicates the entire image to large biochemical systems such as human metabolic pathways.

**Results:**

Our work is inspired by concepts from urban planning since we create a visual hierarchy of biological pathways, which is analogous to city blocks and grid-like road networks in an urban area. We automatize the manual drawing process of biologists by first partitioning the map domain into multiple sub-blocks, and then building the corresponding pathways by routing edges schematically, to maintain the global and local context simultaneously. Our system incorporates constrained floor-planning and network-flow algorithms to optimize the layout of sub-blocks and to distribute the edge density along the map domain. We have developed the approach in close collaboration with domain experts and present their feedback on the pathway diagrams based on selected use cases.

**Conclusions:**

We present a new approach for computing biological pathway maps that untangles visual clutter by decomposing large networks into semantic sub-networks and bundling long edges to create space for presenting relationships systematically.

**Electronic supplementary material:**

The online version of this article (10.1186/s12859-019-2779-4) contains supplementary material, which is available to authorized users.

## Background

Due to the technological and scientific progress, we see a tremendous increase in the knowledge and the amount of collected data in the area of molecular biology and biochemistry over the past years, and computational tools play a major role in this development. One example of increasingly investigated and abundant data are metabolic pathways, i.e., network structures of molecular interactions of biological systems. Collections of such pathways form more complex and hierarchical biological networks, and their careful analysis and understanding are important aspects for many life sciences researchers. Research efforts provide new experimental results, which expand the known networks or require modifications and revisions of previous data. Various initiatives and public databases exist to maintain and curate this growing set of biological network data.

An important step for researchers to make sense of such large networks of biological pathways is to explore visualizations such as pathway diagrams and network layouts, and use them to communicate their respective scientific results in the context of larger biological networks. Automatic network layout algorithms thus become indispensable in the sense that manually creating diagrams of large networks is a very time-consuming if not impractical task, especially considering that the underlying data may change frequently and require permanent layout updates. Sometimes even drastic layout changes are needed. For example, glucose is traditionally considered as a fast supply of energy, while it is nowadays demonstrated that it also affects cancer metabolism [1]. A manually created, static pathway diagram cannot be easily revised to incorporate such an up-to-date information, and pathway designers would need to deliberately move the glucose to a new position based on its changed functionalities. Moreover, as there are several independently managed pathway databases, visualization tools are needed to assist scientists in investigating and understanding biological relationships across multiple databases.

While several general-purpose network layout algorithms exist, most of them are not specifically designed or particularly suitable for drawing biological pathways. This is because in a pathway diagram, detailed relationship information and the corresponding hierarchical grouping structures are expected to be clearly presented simultaneously for analysis and educational purposes [2]. As consequence, biologists still use the few existing high visual quality hand-drawn pathway maps, in order to retrieve the entire image of the roles of chemical components in the network. One example regards human metabolic pathways, which are among the most studied complex pathways and which have been collected by several leading community-driven databases [3, 4]. In 2013, *Recon 2* [5], the most comprehensive metabolic reconstruction that is applicable to computational modeling, was released. It includes about 5063 metabolites and 7440 reactions and has been used to identify reasons and treatments for diseases. Three years later, a hand-crafted pathway map has been integrated with this reconstruction to allow users to explore existing gene expression patterns together with the entire metabolic network. Scientists used this map to figure out how drugs could possibly affect our physiological balance in order to achieve certain treatment effect [4]. This map was created by five undergraduate biochemists in over 20 months by manually reworking on the layout and fixing the errors based on the information in the latest literature. This requires tedious rerouting tasks and still leads to some layout inconsistency due to the decisions made by different collaborators. Other popular metabolic pathway maps, such as *KEGG pathway maps* [3], *Roche Biochemical Pathways* [6], *WikiPathways* [7, 8], and *BRENDA Overview Map* [9, 10] are all manually drawn to achieve their high visual quality, while revisions of them always took months. An automatic layout approach facilitates this drawing process. Recently, *Reactome*, a community-operated knowledge base of biomolecular pathways, has incorporated an automatically generated overview map. It relies on dynamic navigation to assist users in exploring various sub-diagrams [11]. Although their overview layout is computed using a conventional radial network layout algorithm, strong domain knowledge and experience are needed for correct zoom and pan operations. Moreover, tasks based on interaction can come with a time trade-off, since finding particular labels by further exploring an abstract node could consume more time than working with a single layout that has all sufficient information [12].

The aforementioned pathway maps have the disadvantage that biologists need to explore several different maps to build their mental models and knowledge, as each relevant database has its own associated pathway map. Further, there is a high cognitive load to adjust one’s mental map whenever a new version of a pathway map is released. This is because search performance can be facilitated most robustly when objects are tied to spatial locations consistently [13]. From all the above listed reasons it becomes clear that creating high-quality biological pathway layouts automatically (as well as manually) is a very challenging problem. Consequently, in 2017, the annual contest of the International Symposium on Graph Drawing and Network Visualization [14] asked the network visualization community to compete for the best layout of the human metabolic pathway network. However, due to the network’s size and complexity, only one layout produced by an aggregation-based technique was submitted, which is another indicator of the difficulty to automate the task of creating meaningful biological network layouts.

**Fig. 1:**
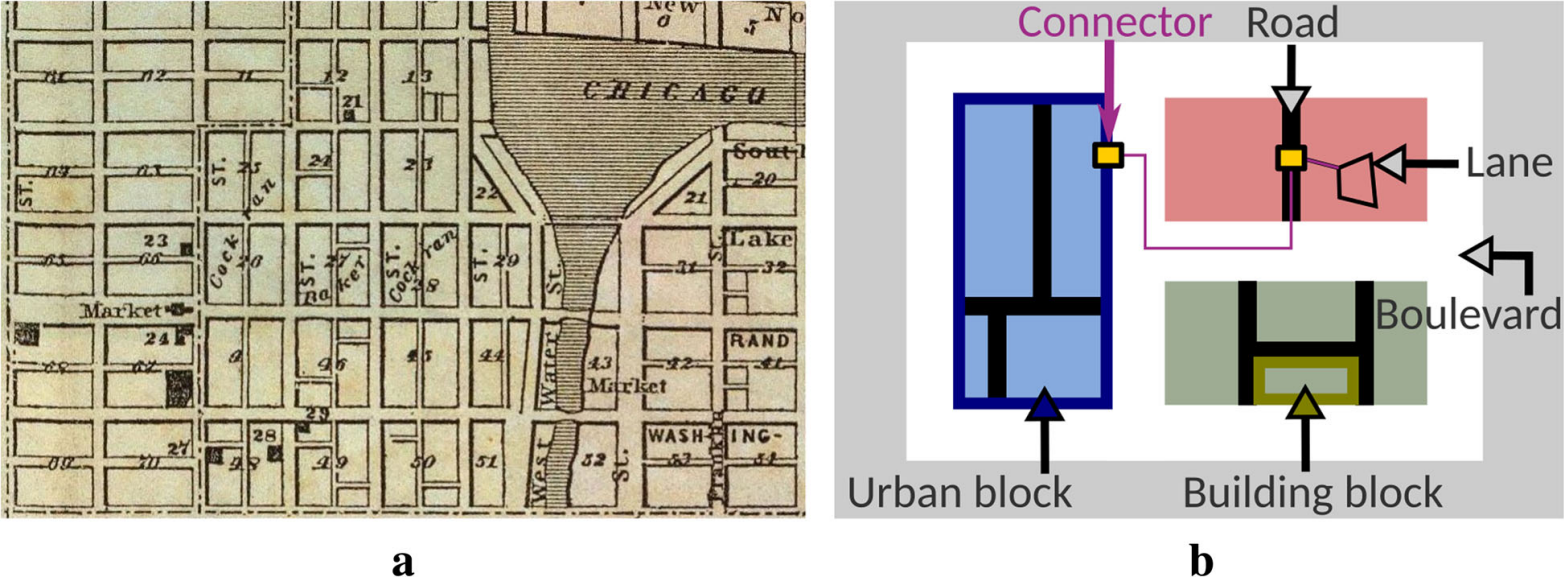
Examples of urban maps, where **a** depicts a map of Chicago in 1857, and **b** shows an abstraction of an urban map

Metabopolis, the new method presented in this paper is the first fully automatic approach for scalable visualization of biological pathways, aiming to combine hierarchical overview with fine detail of individual reactions in order to produce layouts meaningful to the scientific community. The main challenge is the large number of nodes (metabolites) that are heavily interconnected via chemical reactions and how to route the large number of edges without cluttering the pathway map or distracting the user’s attention. Our work is inspired by concepts from urban planning since we create a visual hierarchy of biological pathways, which is analogous to the specification of city blocks and grid-like road networks in an urban area. This structure is considered as the best to have strong mutual connection between neighbors and distribute the traffic density to enhance the sustainability of cities [15]. A typical example is shown in Fig. 1a. We adopt this urban planning concepts and group the underlying hierarchical structures of pathway datasets into multiple rectangular blocks and route edges schematically on the grid of gaps between blocks in order to have avoid clutter and present both low-level and high-level information. Here, low-level information refers to directional or bidirectional relationships between pairs of chemical components and high-level information refers to classified functionalities of these components. Figure 1b shows an abstraction of our maps, where categories are restricted using urban blocks (red, blue, and green rectangles), and sub-graph components are placed in building blocks within each category. The gaps between rectangles serve as boulevards and roads for routing edges to reduce visual complexity.

This is accomplished by automatically creating a graph skeleton together with a possible manual adjustment to guide users’ design decisions followed by a three-step optimization approach computing the final network layout. In the optimization, we first partition the map domain into multiple sub-blocks, then construct the network inside each block, and finally build the corresponding pathway connectivity by routing inter-block edges based on the corresponding context hierarchy.

**Fig. 2:**
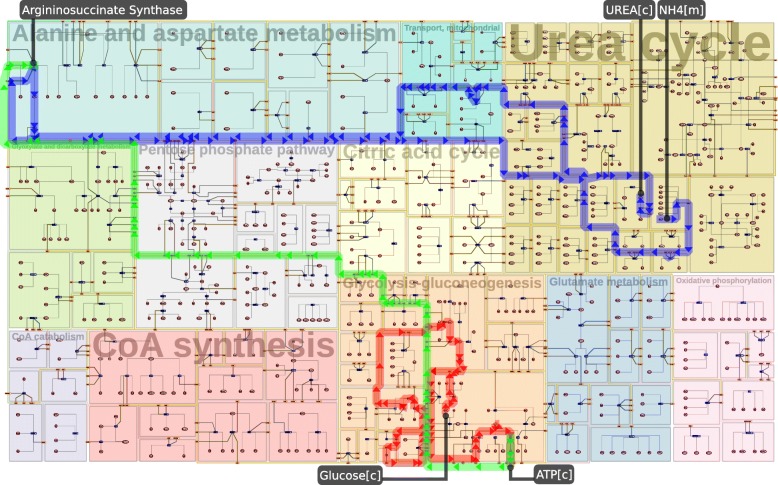
An example of major pathways in human metabolism, including eleven categories highlighted in differently colored blocks. The red route indicates a path for cytoplasmatic oxidation of glucose in cytoplasm in order to obtain ATP (energy), and the blue route shows how humans transform ammonia to urea to eliminate the toxic ammonia in the *Urea Cycle*. Our visualization shows that the first procedure only occurs in *Glycolysis Gluconeogenesis* (orange block) locally, while the chemical components globally move across multiple categories (e.g., mitochondria to cytoplasm through the transport pathways) in the second process. The green route further highlights how the energy generated from the glucose oxidation comes to support catalyzing the urea synthesis. Users can simultaneously read the local and global information using the diagram generated by our system

Figure 2 presents an example of a diagram created by Metabopolis, which includes eleven major pathways presented in different colors. One of the main mechanisms to produce energy in human body is the *Glycolysis* process (orange), where the red route shows the set of reactions for the biological transformation of glucose into pyruvate. This happens together with the releasing of high-energy molecules of ATP, the universal energy currency used to drive more biological reactions (see the green route). ATP then comes to the blue route to synthesize urea. Our diagram allows us to read this network by visually restricting information into rectangular blocks to facilitate a better understanding on the local and global contexts of the network. Our technique enables us to compute the pathway diagram of the entire human metabolism (see Fig. 11), which has never been achieved in a comparable quality using conventional network layout techniques.

The remainder of this paper is structured as follows: “Related work” section summarizes relevant related work. In “Overview of Metabopolis” section, we explain the introduced design criteria for large pathway diagrams together with a summary of our proposed system framework. The technical details are presented in “Urban block construction”, “Intra-block network layout”, and “Inter-block network layout” sections. “Urban block construction” section explains the steps for computing the floorplan layout of the hierarchical grouping structure. “Intra-block network layout” and “Inter-block network layout” sections present the intra- and inter-block network layout, respectively. Our implementation is detailed in “Implementation and enhancement” section followed by the use cases and discussion in “Experimental results, evaluation, and discussion” section. We conclude this paper and refer to future directions in “Conclusion and future work” section.

## Related work

In this section, we conduct a brief survey on the most relevant related topics of this work, including pathway visualization, space partitioning approaches, and map-based network visualization.

### Pathway visualization

Since new biological pathways are unceasingly investigated and added to pathway databases, pathway visualization [16–19] has developed a variety of alternative representations to support researchers in reasoning about pathways. Murray et al. [2] summarized common visualization tasks for the analysis of biological pathway data. They consider relationship tasks as the most essential tasks in their study. Existing general-purpose network visualization tools for highlighting hierarchical relationships are not that suitable for biological pathways since low-level representation may be aggregated to show the underlying grouping structures [20, 21]. Therefore, although several network visualization techniques have been developed for this purpose, researchers still rely on the manually designed pathway maps provided from biological databases [3, 4]. Pathway editors such as CellDesigner [22], SBGN-ED [23], and Newt [24], and network analysis tools such as Cytoscape [25] provide functionalities for dynamic pathway analysis, while the layout problem is still resource-consuming, especially for graphs with more than 500 nodes. This is because the underlying graph layout techniques are often developed for general purposes and cannot be easily applied to large biological networks.

The investigation of biological pathway visualization has mainly two directions, including drawing fine small pathways and aggregating detailed pathways to visualize high-level information. Several research works focus on visually pleasing and well readable layout of small biological networks, including rebuilding well-known KEGG maps [26], overlaying omics data [27], aligning nodes on grids [28], and the most popular forced-directed and hierarchical layouts summarized by Bachmaier et al. [19]. Other works relied upon strong user interactions on hand-drawn large but static maps [29]. Interactions such as semantic zooming [30] and aggregation [31, 32] have been investigated to analyze large networks. Although interactions have been important tools to facilitate users’ capabilities to understand large datasets, it has also been studied that interactive activities during the analysis process may increase time for accomplishing simple connectivity tasks [33]. Nevertheless, interaction is definitely a valuable way to support analytical processes, where users can expand and collapse the visualization to retrieve their target of interests. Unfortunately, neither of the aforementioned directions resolves the difficulty on the communication of knowledge since researchers always need to rebuild their mental image to various maps introduced by different databases. Compared to the aforementioned interaction techniques, our approach provides an alternative solution to biologists. This is because we introduce a graph skeleton to assist biologists to design their pathway diagram, and introduce orthogonal layout and edge routing to maintain the readability of low-level and high-level relationship information.

### Space partitioning and planning techniques

Space partitioning algorithms using techniques based on *Voronoi diagrams* [34], *treemaps* [35, 36], and *floor planning* [37] subdivide a space into several disjoint subregions and are often used to assign the screen space in information visualization. Among these, *floor planning* algorithms have been well investigated in *very large scale integration (VLSI)* design to generate constrained high quality chip layout [37, 38]. In our implementation, we select *floor planning* algorithms as the basis of our optimization process due to their flexibility in attaching user defined rectangles.

For example, Merrell et al. [39] developed an approach to automatically design room layouts trained on real-world data and Ma et al. [40] calculated a room plan based on a planar graph specified by game designers. Both methods introduced configuration space techniques to further constrain object placement during the optimization process. The more high-level requirements designers provide, the higher the computational time needed for the stochastic optimization. In our approach, we introduce several constraints to control appropriate block placement, which reproduce results similar to hand-drawn pathway maps and limit the search space for our optimization process.

### Map and network visualization

Clustered network visualization has been studied widely [41–43], but those works either focus on small compound graphs or aggregate directed edges due to the scalability of the approach. Rather we chose a map metaphor for Metabopolis because maps are one of the most popular visual representations to describe object relationship and relative positions within a certain space [44]. A pioneering work of visualizing graphs as maps, has been done by Gansner et al. [45, 46], where they partition the map domain using a Voronoi diagram and a force-directed algorithm to draw subgraphs in each Voronoi cell. Several works applying the map metaphor were published subsequently, e.g., topographic maps of clustered graphs [47], maps of computer science [48], and GraphMaps [49]. As pathway map designers often do, simplifying edge structures is also studied in the context of map-based visualizations, which include hierarchical Manhattan layout [50], road maps [51, 52], and metro maps [53]. Orthogonal graph layout is a specific and well-studied type of schematic layout, where edge segments are limited to horizontal and vertical directions [54]. More recently, high-quality compact orthogonal layout of small graphs was the focus of studies [55, 56]. In our layouts, we decompose a large metabolic network into smaller sub-graphs to employ orthogonal layout algorithms such as HOLA [55] or yFiles [57] compact layout for visualizing pathway relationship in detail, which is the edge style favored by pathway designers [3].

## Overview of Metabopolis

A good biological pathway map should be an easy-to-read visual representation of the molecules in a cell and their relations through biochemical reactions in detail together with their corresponding hierarchical grouping structures [2]. Although, this criterion is expected to be the leading criterion for the design of pathway diagrams, general graph drawing criteria should also be taken into consideration. Notably, these maps should preserve the mental images of biologists, which also affects users’ memorability of the content [58]. Within the biological context, reactions are often expressed using an arrow →, where the reactants are placed on the left and the products are on the right. We can model a biological pathway network using a bipartite directed graph *G*=(*V,E*), where *V*=*M*∪*R*. The nodes in *M* are the metabolites and the nodes in *R* represent the reactions. A directed edges *e*∈*E* represents the involvement of metabolites in reactions as either reactant or product. Note that each metabolite *v*_*m*_∈*M* can be involved in multiple semantic categories *c*(*v*_*m*_)⊂*C* (e.g., a subsystem defined in a standard ontology, a compartment of the cell, etc), while each reaction *v*_*r*_∈*R* belongs to a unique category *c*(*v*_*r*_)∈*C*. Moreover, biochemical reactions can be either bidirectional (e.g., 6*CO*_2_+6*H*_2_*O*⇔*C*_6_*H*_12_*O*_6_+6*O*_2_) or unidirectional (e.g., *C*_3_*H*_8_*O*_3_ + 3 *CH*_3_(*CH*_2_)_6_*COOH*→*C*_55_*H*_98_*O*_6_ + 3*H*_2_*O*), which is essential for a comprehensive understanding of physiological processes.

**Fig. 3:**
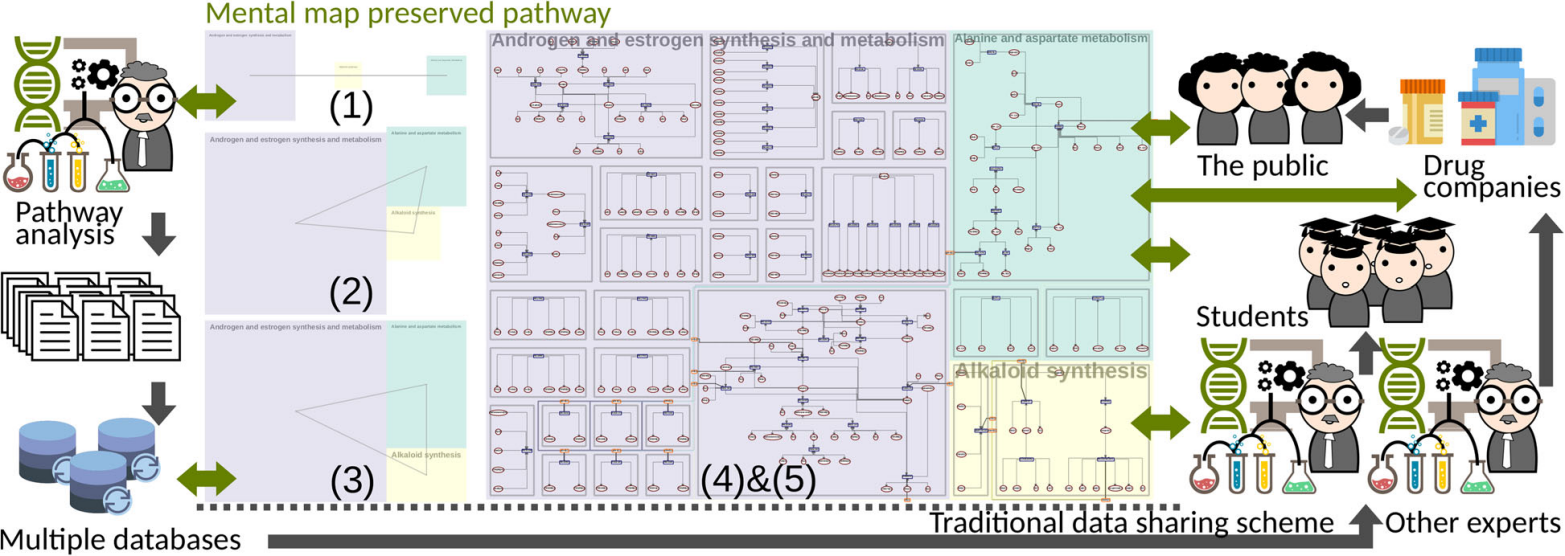
Our visualization framework to support pathway analysis, including (1) constructing a user-specified connectivity graph, (2) overlap-free rectangle placement, (3) maximizing screen space, (4) constructing orthogonal layout of each subgraph and (5) highlight relationships among different categories

Metabopolis provides a new type of pathway diagram using an urban map metaphor to bridge the gap between different hand-drawn pathway maps while preserving the readability from low to high levels. Figure 3 depicts how automatic pathway maps can serve as key media that allow users to share and communicate their data. Users can automatically create maps with similar category alignment and mutually share them. The gap is closed by turning one-way (black arrows) to round-way (green arrows) information delivery by enabling the entire community to interact with the same data.

To accomplish this goal, we have first investigated all well known hand-drawn pathways, and summarize the challenges **(A1-A3)** of the existing pathway layouts as follows:

- **(A1)** Preserving a user’s mental map of the diagram or customizing the network layout with updated data are not easy. Domain experts need to adapt to different layouts and map between different mental models in order to use their knowledge consistently.
- **(A2)** No clutter management strategy exists to control the visual density between global and local context. Metabolites involved in many reactions are often high-degree nodes, some of which can be significant (e.g. *ATP*, the energy currency of the cell) and some can be less informative (e.g. water molecules) to the scientists.
- **(A3)** A readable visual hierarchy is missing to present low-level and high-level relationship information simultaneously. Directed/bidirected edges and categories are crucial to identify the roles of chemical components in the physiological system.

These three major challenges are tackled by our pathway layout algorithm, and each of them will be solved using three types of networks, a graph skeleton *G*_*C*_, an extended pathway network *G*_*D*_, and two flow networks *G*_*M*_ and *G*_*N*_, respectively.

Our strategy to cope with **(A1)** is to introduce a graph skeleton *G*_*C*_ used to preserve or customize the relative positioning of urban blocks in *B*_*C*_, which corresponds to the drawing area reserved for each category *c*∈*C*. The category here can refer to any semantic category defined in the pathway ontology, where we use the biological subsystems as a proof of concept in our system. The graph skeleton is then defined as *G*_*C*_=(*B*_*C*_,*E*_*C*_), where each block *b*_*c*_ in *B*_*C*_ is a rectangular node for the corresponding category and each edge *e*_*c*_∈*E*_*C*_ indicates the connectivity between blocks. The initial position of a block and the connectivity between a pair of blocks can be computed automatically using our system or refined by the users. This allows us to automatically place blocks sharing more chemical components close to their neighborhood to reduce long edges across the entire map domain.

**Fig. 4:**
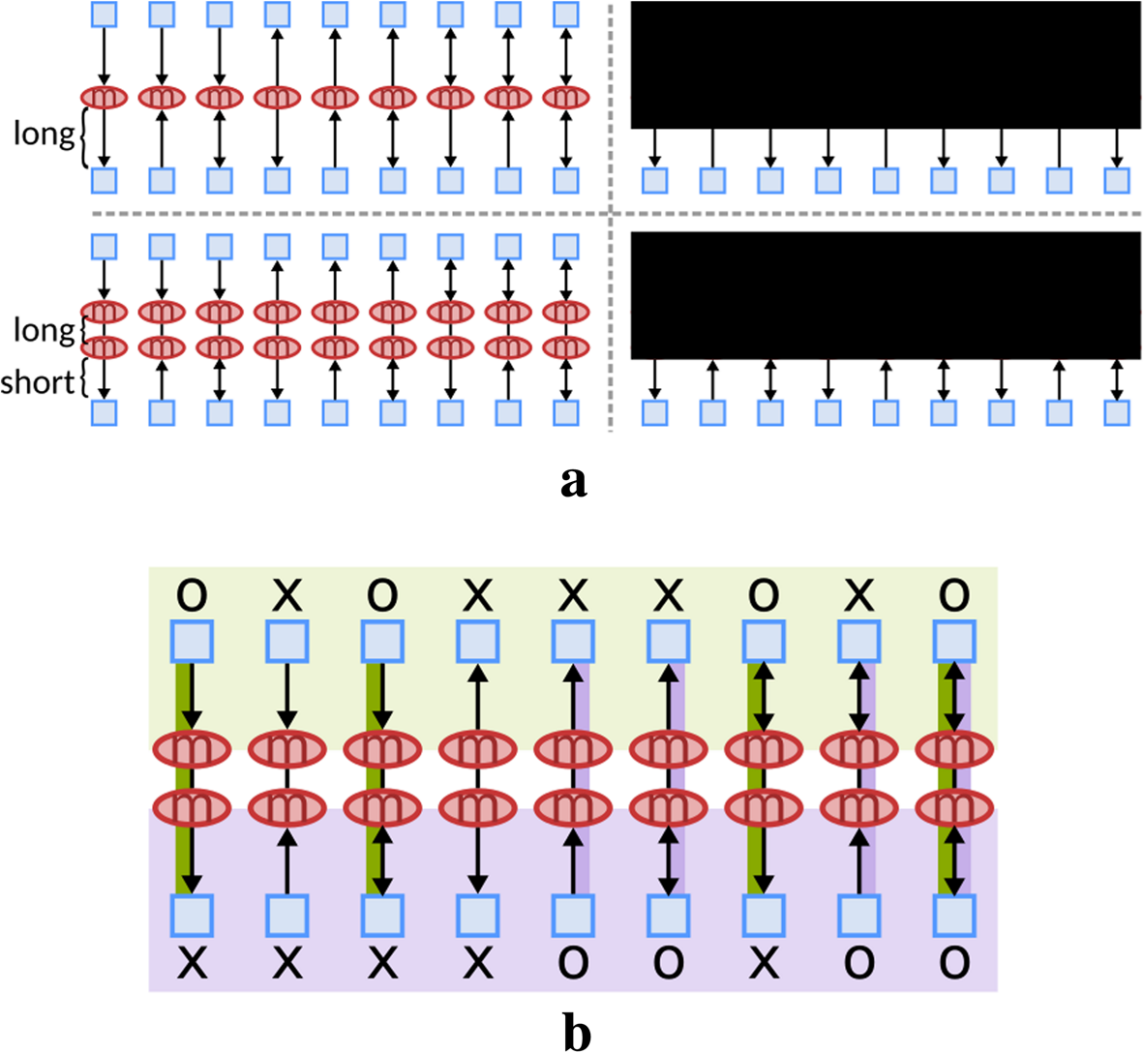
Our design for long edges, including **a** a long directed path decomposition and **b** the corresponding color coding of discriminating types of communication

Dealing with **(A2)** is achieved by the duplication of the same high-degree or user-specified unimportant metabolites that are connected by a secondary layer of edges. This provides users an opportunity to discriminate between important metabolites such as glucose and unimportant ions such as water. All original and duplicated nodes are collected in *V*_*D*_, and corresponding edges will be stored in *E*_*D*_ to form our new network *G*_*D*_ for visualization. Even though node duplication reduces edge density of a graph, several long edges may occur in the layout. Therefore, we decompose long directed edges into a set of directed and undirected edges (see Fig. 4a) so that we can bundle undirected edges, which are less informative to control the visual density between global and local context.

This allows us to visually discriminate high-degree nodes into two types. The first type of metabolites are unimportant (as specified by domain experts), and are fully duplicated in Metabopolis. The second ones are those metabolites serving as connectors, which are significant targets of interests for biologists since they are connected to reactions having different semantics and should not be easily duplicated in the visualization. Figure 4b shows an example of this design between two categories (green and purple). We use colors to highlight the roles of metabolites between each pair of categories, and there are all nine possible combinations of the roles of the metabolites between two categories. Take the first column for example, the green path indicates that there exists a product metabolite *m* from the green category that serves as a reactant in a reaction in the purple category, but no inverse reaction is allowed. Although the third column has the same color coding as the first one, the output arrow indicates that this product metabolite serves as a reactant in another category but not the green one. With this design, we can bundle long undirected edges along the boundary of blocks, while not sacrificing the clarity of the edge representation.

Finally, to deal with **(A3)**, we create compact orthogonal drawings for sub-networks within each category and bundle undirected edges along the boundary of the blocks to achieve a readable visual hierarchy of our maps. Note that map metaphors have been proved as effective designs to visualize graphs and clusters [45, 46], because of the geospatial positions of objects and their corresponding connections can be shared between users as well as the general familiarity of maps among the public. Urban maps are a specific type of map used to visualize buildings and roads in a city. These objects are often simplified to certain geometric shapes such as lines, rectangles, and squares, in order to facilitate the general understanding of graphical notations on maps. We follow this example by restricting category information to be represented as rectangles and by aligning objects to underlying grids in our diagrams. We align vertices and edges on grids because this is a common strategy employed in many hand-drawn pathway diagrams [3, 4].

Figure 3 shows the pipeline of our algorithm, which consists of five steps. (1) We first automatically construct a spanning subgraph of the categories based on the frequency of inter-category edges for guiding block placement. In this step, users are also allowed to edit the graph under certain constraints. (2) Afterwards we apply a constrained floor-planning algorithm to attach strongly-connected categories along a shared boundary and produce an overlap-free block placement. (3) Next, since the number of metabolites in each category determines the block size, we adjust the size of these blocks to optimize the screen space partitioning. (4) Within each category block, we use an orthogonal network layout to place and align metabolites and reactions on a grid. (5) Finally, we construct an auxiliary flow network to disperse flows to optimize edge routing for connecting identical metabolites. Steps (1)-(3) will be detailed in “Urban block construction” section and steps (4)-(5) will be described in “Intra-block network layout” and “Inter-block network layout” sections.

## Urban block construction

In this section, we introduce how the map domain is partitioned into multiple sub-blocks, while aligning blocks with strong connectivity as neighbors using a graph skeleton. We formulate the computational problem as a *mixed-integer programming* (MIP) model, to find a globally optimal solution.

### Graph-based skeleton for guiding block placement

Biologists usually investigate a specific protein or gene, a set of specific pathways or more recently, due to the improvement in pipelines for analysis of high-throughput data, entire metabolic networks. In all cases, they are interested to see the context of the results generated under their experiments, which leads to comparison tasks on relationship between similar sets of chemical components. Thus, a pathway diagram with categorical information highlighted allows biologists to compare the relationship within one category and between each other. We thus propose a graph-based skeleton *G*_*C*_, a spanning subgraph of the category connectivity graph, to optimize the placement of entire blocks. This is because connecting all pairs of blocks sharing some reactants or products will create a nearly complete connectivity graph, and it is more important that blocks having dense connection in-between should be placed next to each other.

We extend the conventional *floor-planning* problem by adding additional alignment constraints to guarantee the connection of all sub-blocks. This is achieved by optimizing the block positions according to the connectivity of *G*_*C*_. Note that planar graphs have been previously used to guide users for designing a room layout [39, 40] guaranteeing a doorway continuity. However, our graph skeleton should not only serve this purpose, but should also present users with a clear information whether the designed graph will produce a solvable result. All types of planar graphs are not sufficient for the block placement in our case.

**Fig. 5:**
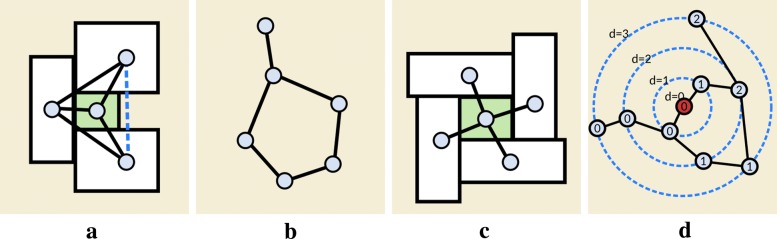
Examples of building our graph skeleton, including **a** a *K*4 graph, **b** a chordless graph, **c** a degree 4 star graph, and **d** an appropriate order for initial crossing-free layout

The skeleton graph *G*_*C*_ provides an important instruction here because typically the category graph is very dense and not all edges can be represented as block adjacencies. Obviously, only planar graphs can be represented by touching rectangles, but even some planar graphs cannot be represented. If they have separating triangles (see *K*_4_ in Fig. 5a), it is known to fail [59]. Inspired by the semantic word cloud technique [60], which also aims to optimize the placement of touching rectangles, we know that if the skeleton is a graph with only disjoint cycles (see Fig. 5b), a corresponding floor plan always exists. Moreover, Fig. 5c depicts another extreme case of the graph skeleton, where a node with degree larger than four would also produce an undesired layout since we cannot attach another big block to the green block. In summary, we design our graph skeleton *G*_*C*_ under constraints: the graph (1) has to be planar, (2) has to contain only edge-disjoint cycles, and (3) has maximum node degree four. This will create a so-called chordless planar graph (see Fig. 5b), which usually contains long chains. Note that we do not aim to get a maximally dense planar graph, but rather one that maintains a sufficient degree of flexibility. Our system automatically generates *G*_*C*_ by expanding a maximum-weight *spanning graph*. This is done by first sorting edges in descending order and greedily include a pair of blocks with maximum weight value as long as the graph remains planar, chordless, and with maximum node degree at most four. The weight value of an edge is defined by the frequency of metabolites appearing in both blocks. Once this basic skeleton is computed, users can further edit *G*_*C*_ to match their specific aims, personalizing the network by adding or removing inter-block connectivities. Metabopolis then automatically initializes the graph using a new crossing-free layout algorithm by default. This is done by sorting the nodes having the same topological distance to the geodesic center on each branch and place nodes on concentric circles according to their distance (see Fig. 5d).

### Constrained floor-plan problem

**Fig. 6:**
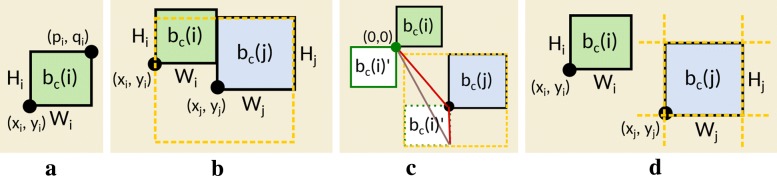
Illustrations of our mathematical constraints, including **a** block representation using Chebyshev distance, **b** alignment of blocks along boundaries, **c** configuration space by Minkowski sum, and **d** overlap free condition

Once we have the graph skeleton, we are ready to place blocks based on its connectivity. In this subsection, several hard and soft constraints to place blocks of pathway subsystems based on their connectivity or desired positioning will be introduced to find an appropriate layout in our MIP model. *Mixed-integer programming* (MIP) is an optimization technique where variables can be either integers or real numbers that are subject to a set of constraints. The constraints can be linear equalities or inequalities, together with a linear objective function to be optimized. A globally optimal solution for a MIP model can then be computed using specialized MIP solvers such as CPLEX or Gurobi. In this framework we can model hard constraints and soft constraints to fully or partially fulfill aesthetic criteria for the layout, while seeking for the best solution under the employed conditions. Initially, we assign a rectangular area *b*_*c*_∈*B*_*C*_ proportional to the amount of reactants and products in each category as a reserved region for drawing, so that we can apply aesthetic criteria to compute the desired space for enhancing pathway readability. Figure 6a depicts how a block *b*_*c*_(*i*) is formulated in our system, with two reference points (*x*_*i*_,*y*_*i*_) and (*p*_*i*_,*q*_*i*_) referring to its bottom-left and top-right corners respectively, together with its corresponding width *W*_*i*_ and height *H*_*i*_. To achieve our strategy to **A(1)**, we incorporate several hard (CH1–CH4) and soft (CS1–CS3) constraints in MIP model, which are summarized as:

- (CH1) **Block-block attachment:** The two blocks connected with an edge must be placed next to each other.
- (CH2) **Overlap-free block placement:** The placement must be overlap-free.
- (CH3) **Pairwise relative positioning:** Mutual relative positions of blocks as specified by the graph skeleton are preserved.
- (CH4) **Barycenter preservation:** Relative positions between the barycenter of a cycle and its end nodes are preserved.
- (CS1) **Compact layout:** The layout should be compact.
- (CS2) **Expected aspect ratio:** The layout should adhere to the desired aspect ratio.
- (CS3) **Long shared boundary:** Attached blocks should have long shared boundaries.

#### Block-block attachment constraints (CH1)

This constraint allows us to attach two neighboring blocks from the graph skeleton so that each pair of blocks will have exactly one shared boundary in the output, as the two blocks *b*_*c*_(*i*) and *b*_*c*_(*j*) shown in Fig. 6b. The yellow dotted rectangle here indicates the configuration space for *b*_*c*_(*j*), to represent all possibilities of placing (*x*_*i*_,*y*_*i*_) along *b*_*c*_(*j*) so that the two blocks are in contact but do not overlap [61, 62]. This is done by reflecting *b*_*c*_(*i*) at its reference point on (0,0) (see Fig. 6c), computing the Minkowski sum (*b*_*c*_(*i*)^′^+*b*_*c*_(*i*)=*a*+*b*|*a*∈*b*_*c*_(*i*)^′^,*b*∈*b*_*c*_(*j*)) of two blocks *b*_*c*_(*i*) and *b*_*c*_(*j*), and computing the convex hull to extract the polyline configuration space.

For each pair of connected blocks, we decompose the configuration space of *b*_*c*_(*j*) into multiple line segments *L*(*r*):*Ax*+*By*+*C*=0 (*r*=1,…,*k*) and force the reference point (*x*_*i*_,*y*_*i*_) of *b*_*c*_(*i*) to settle on one of these segments. For each *L*(*r*), the constraint to place (*x*_*i*_,*y*_*i*_) on the corresponding configuration space is defined as:

1 $$ \alpha_{L(1)}(i,j)+\alpha_{L(2)}(i,j)+...+\alpha_{L(k)}(i,j) \geq 1, \text{ and } \\  $$

2 $$ \begin{aligned} y_{i} - y_{j} &\leq - A/B \cdot (x_{i}-x_{j}) -C/B + (1-\alpha_{L(r)}(i,j)) \cdot M \\ y_{i} - y_{j} &\geq - A/B \cdot (x_{i}-x_{j}) -C/B - (1-\alpha_{L(r)}(i,j)) \cdot M \\ x_{i} - x_{j} &\leq X_{max} + (1-\alpha_{L(r)}(i,j)) \cdot M \\ x_{i} - x_{j} &\geq X_{min} - (1-\alpha_{L(r)}(i,j)) \cdot M \\ y_{i} - y_{j} &\leq Y_{max} + (1-\alpha_{L(r)}(i,j)) \cdot M \\ y_{i} - y_{j} &\geq Y_{min} - (1-\alpha_{L(r)}(i,j)) \cdot M, \\ &\vdots \end{aligned}  $$

where *α*_*L*(*r*)_(*i,j*) for *r*=1,…,*k* are binary variables and *M* is a large constant used to automatically validate and invalidate the set of the constraints to place (*x*_*i*_,*y*_*i*_) on *L*(*r*) in the MIP model. Note that (*x*_*i*_,*y*_*i*_) and (*x*_*j*_,*y*_*j*_) are reference points of block *b*_*c*_(*i*) and *b*_*c*_(*j*), and *A*, *B*, and *C* are constants precomputed from line *L*(*r*). (*X*_*min*_,*X*_*max*_) and (*Y*_*min*_,*Y*_*max*_) indicate the lower and upper bounds of each *L*(*r*) along *x* and *y* axes, respectively. Since *M* needs to be larger than all coordinates of *x*_*i*_ and *y*_*i*_, we define our *M* as $\sum _{i \in V}{(W_{i}+H_{i})/2}$. We also use *k*=4 by default since a rectangle has four boundaries.

#### Overlap-free block placement constraints (CH2)

Generation of *floor plans* is a challenging task because the layout must be overlap-free. Figure 6d depicts an example of this constraint, where block *b*_*c*_(*i*) needs to be placed outside one of the boundaries of block *b*_*c*_(*j*), and therefore is formulated as:

3 $$ \beta_{\text{left}}(i,j)+\beta_{\text{bottom}}(i,j)+\beta_{\text{right}}(i,j)+\beta_{\text{top}}(i,j) \geq 1, \text{ and } \\  $$

4 $$ \begin{aligned} x_{i} + W_{i} &\leq x_{j} + (1-\beta_{\text{left}}(i,j)) \cdot M \\ y_{i} + H_{i} &\leq x_{j} + (1-\beta_{\text{bottom}}(i,j)) \cdot M \\ x_{i} &\geq x_{j} + W_{j} - (1-\beta_{\text{right}}(i,j)) \cdot M \\ y_{i} &\geq x_{j} + H_{j} - (1-\beta_{\text{top}}(i,j)) \cdot M. \\ \end{aligned}  $$

Note that we again introduce binary variables *β*(*i,j*) to validate and invalidate one of the four conditions, and *M* is the same large value from Eq. (2) reused in Eq. (4).

#### Pairwise relative positioning constraints (CH3)

**Fig. 7:**
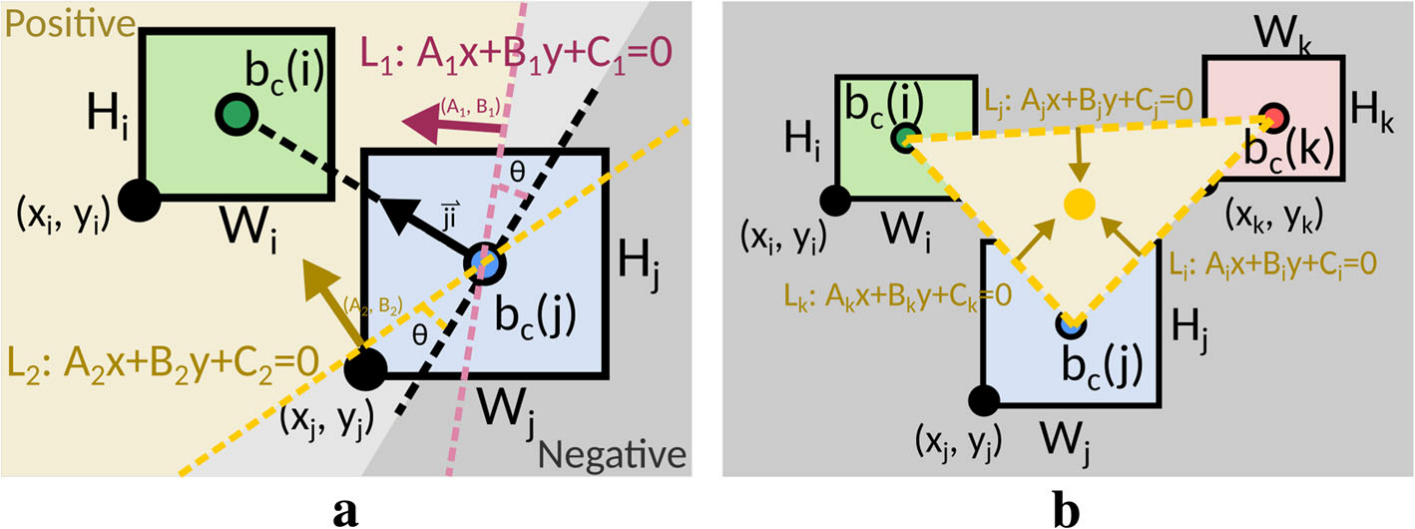
Illustrations of relative positioning constraints, including **a** preservation of spatial relationship and **b** barycenter of a triangle face

This relative position constraint is used to maintain the spatial relationship between each pair of blocks, which helps preserving the mental map from the diagram created previously, as well as limiting the search space in the model. Figure 7a depicts an example of such a constraint, where the map domain is divided into the positive side (*A*_*n*_*x*+*B*_*n*_*y*+*C*_*n*_>0) and the negative side (*A*_*n*_*x*+*B*_*n*_*y*+*C*_*n*_<0) and this condition needs to be preserved after the optimization [63]. To control this constraint, we newly introduce an angle *θ* to generate two border lines *L*_1_ and *L*_2_ that are used to designate feasible region for block placement (yellow region for *b*_*c*_(*i*) in Fig. 7a). Note that the constant values *A*_*n*_,*B*_*n*_, and *C*_*n*_ are computed from the initial coordinates of *b*_*c*_(*i*) and *b*_*c*_(*j*), where we rotate the normal vector of $\overrightarrow {ji}$ by the angle *θ* clockwise and counterclockwise. Since we define (*A*_*n*_,*B*_*n*_) as unit normal vector, which satisfies |*A*_*n*_|^2^+|*B*_*n*_|^2^=1 of lines *L*_*n*_, so that we can compute the signed distance *D*_*n*_ between *b*_*c*_(*i*) and *L*_*n*_ simply by inner product. In other words, if the block *b*_*c*_(*i*) is located on the positive side originally then it will be forced to stay on the same side in the computed floorplan. The constraint is formulated if *D*_*n*_>0 or *D*_*n*_<0, respectively as:

5 $$\begin{array}{@{}rcl@{}}  \scriptsize  \begin{array}{l} A_{n}(x_{i}+\frac{W_{i}}{2} - x_{j}-\frac{W_{j}}{2}) + B_{n}(y_{i}+\frac{H_{i}}{2} - y_{j}-\frac{H_{j}}{2}) \geq |D_{n}|, \\ A_{n}(x_{i}+\frac{W_{i}}{2} - x_{j}-\frac{W_{j}}{2}) + B_{n}(y_{i}+\frac{H_{i}}{2} - y_{j}-\frac{H_{j}}{2}) \leq -|D_{n}|. \end{array} \end{array} $$

#### Barycenter preservation constraints (CH4)

In most of the cases, pairwise relative positioning constraints will also preserve the planar embedding of the network, while in some extreme cases such as a small block connected to two large ones, will break these rules since the border lines *L*_1_ and *L*_2_ are close to parallel. To solve this, we introduce another constraint that restricts the barycenter of a cycle inside the cycle after optimization (see the yellow cycle in Fig. 7b) to preserve the planar embedding after optimization. The constraint is similar to the *paiwise relative positioning constraints (CH3)*, where we keep the barycenter of all end points of a cycle retaining at the same side as their original position (yellow region in Fig. 7b). Blocks *i*, *j*, and *k* are three blocks composing a triangle face *F*_*k*_, and the yellow point indicates their corresponding barycenter. This constraint is thus revised from Eq. (5) by replacing $x_{i}+\frac {W_{i}}{2}$ with *x*_avg_ and $y_{i}+\frac {H_{i}}{2}$ with *y*_avg_, respectively, where (*x*_avg_,*y*_avg_) is the barycenter of the cycle at initial position.

#### Objective function (CS1–CS3)

Beside the aforementioned hard constraints, we also introduce several soft constraints for better usage of screen space. Our goal here is to find a compact layout (CS1) having expected aspect ratio (CS2) and long shared boundaries between blocks (CS3).

[Compact layout (CS1)] is accomplished by minimizing the objective function *obj*_compact_=*w*_compact_·(*B*_*x*_+*B*_*y*_), where we introduce the upper bounds *B*_*x*_ and *B*_*y*_ to every blocks *b*_*c*_(*i*) by 0≤*x*_*i*_≤*B*_*x*_−*W*_*i*_ and 0≤*y*_*i*_≤*B*_*y*_−*H*_*i*_.

[Expected aspect ratio (CS2)] is done by minimizing the objective function *obj*_ratio_=*w*_ratio_·*δ*, where *δ* is defined as *δ*=|*B*_*x*_−*R*·*B*_*y*_| for the user-specified target aspect ratio *R*. Our default is *R*=4/3.

[Long shared boundary (CS3)] is achieved by minimizing the objective function $obj_{\text {overlay}} = w_{\text {overlay}} \cdot \sum _{e_{ij} \in E_{C} }{ (\gamma _{x}(i,j) + \gamma _{y}(i,j)) }$, where *γ*_*x*_(*i,j*) and *γ*_*y*_(*i,j*) are displacements between pairwise block centers along *x* and *y* axes, which are defined as

6 $$\begin{array}{@{}rcl@{}}  \scriptsize \hspace{0pt} \begin{array}{l} |x_{i} + \frac{W_{i}}{2} - (x_{j} + \frac{W_{j}}{2})| = \gamma_{x}(i,j) \text{, and} \\ |y_{i} + \frac{H_{i}}{2} - (y_{j} + \frac{H_{j}}{2})| = \gamma_{y}(i,j). \\ \end{array} \end{array} $$

Finally, we minimize the sum of three objective terms as follows:

7 $$\begin{array}{@{}rcl@{}}  \scriptsize \begin{array}{l} obj_{\text{floorplan}} = obj_{\text{compact}} + obj_{\text{ratio}} + obj_{\text{overlay}}. \\ \end{array} \end{array} $$

Note that by default, we empirically employ *w*_overlay_=10,*w*_compact_=1000, and *w*_ratio_=1 for the weights in our system.

### Fine block adjustment

Once we have a compact layout for the block placement, we then fine adjust the four boundaries of each block for better utilizing the screen space. This allows us to align block boundaries to avoid bends when routing the edges. The optimization partially includes the aforementioned constraints in “Constrained floor-plan problem” section, together with new constraints for this purpose. We again prepare a left-bottom reference point (*x*_*i*_,*y*_*i*_) for each *b*_*c*_(*i*) but add a right-top reference point (*p*_*i*_,*q*_*i*_) to facilitate adjusting block boundaries. Our hard (CH2–FH2) and soft (CS1–FS2) constraints are summarized as:

- (CH2) **Overlap-free block placement:** As defined previously.
- (FH1) **Minimal block width and height:** The minimum drawing area must be preserved.
- (FH2) **Attached boundary:** Attached boundaries must stay.
- (CS1) **Compact layout:** As defined previously.
- (CS2) **Expected aspect ratio:** As defined previously.
- (CS3) **Long shared boundary:** As defined previously.
- (FS1) **Area maximization:** Drawing area should be maximized.
- (FS2) **Block aspect ratio:** Aspect ratio of each block should not be changed drastically.

#### Minimal block width and height (FH1)

Our objective is to preserve the minimal drawing space computed from the previous step in “Constrained floor-plan problem” section to avoid drastic territory changes. The idea is simple since we assign lower bounds to the width and height of each block. To preserve the minimum block size, the constraints are formulated as *p*_*i*_−*x*_*i*_≥*W*_*i*_ and *q*_*i*_−*y*_*i*_≥*H*_*i*_, where *W*_*i*_ and *H*_*i*_ are the width and height of the block computed previously. Note that this is the constraint employed to guarantee minimal drawing space of each block *b*_*c*_(*i*).

#### Attached boundary constraints (FH2)

We also incorporate hard constraints to retain the shared boundaries computed from the previous optimization. Since we already know the shared boundary between adjacent blocks, the idea here is simple. We force the distance between the shared boundary to 0, while restricting the corresponding upper bounds always locating higher than the lower bounds among the two blocks. These constraints are added by investigating all the shared boundaries along *x* and *y* axes and are formulated as:

8 $$\begin{array}{@{}rcl@{}}  \scriptsize  \begin{array}{l} p_{i} - x_{j} = 0, \, q_{i} - y_{j} > 0, \text{ and}\,\, q_{j} - y_{i} > 0, \text{ if}\,\, |p_{i} - x_{j}| = 0, \\ q_{i} - y_{j} = 0, \, p_{i} - x_{j} > 0, \text{ and}\,\, p_{j} - x_{i} > 0, \text{ if}\,\, |q_{i} - y_{j}| = 0. \\ \end{array} \end{array} $$

#### Objective function (FS1–FS2)

We again introduce several soft constraints for better usage of screen space, including compact layout (CS1), expected aspect ratio (CS2), area maximization (FS1), long attached boundary (FS2).

**[Area maximization (FS1)]** is achieved by minimizing the distance between the block boundaries and the four boundaries of the map domain. This objective is therefore defined as $obj_{\text {area}} = w_{\text {area}} \cdot \sum _{i \in V}{(x_{\text {distL}}(i)+y_{\text {distL}}(i)+x_{\text {distU}}(i)+y_{\text {distU}}(i))}$, where the distance to each of the boundary is defined as *x*_*i*_=*x*_distL_(*i*),*x*_upper_(*i*)−*p*_*i*_=*x*_distU_(*i*),*y*_*i*_=*y*_distL_(*i*), and *y*_upper_(*i*)−*q*_*i*_=*y*_distU_(*i*).

**[Block aspect ratio (FS2)]** is similar to constraint *expected aspect ratio (CS2)*, where we apply the idea to each block. The objective $obj_{\text {blockratio}} = w_{\text {blockratio}} \cdot \sum _{i \in V}{\kappa _{i}}$, is minimized by collecting the corresponding distortion as *κ*_*i*_=|*p*_*i*_−*x*_*i*_−(*q*_*i*_−*y*_*i*_)|.

Finally, we minimize all objective terms by summarizing them as:

9 $$\begin{array}{@{}rcl@{}}  \scriptsize  \begin{array}{l} obj_{\text{adjustment}} = obj_{\text{floorplan}} + obj_{\text{area}} + obj_{\text{blockratio}}. \\ \end{array} \end{array} $$

We empirically employ the same weight value for *obj*_floorplan_, and set *w*_area_=1000 and *w*_blockratio_=100 by default in our system.

## Intra-block network layout

The grid pattern is commonly chosen in urban planning even though its infrastructure cost is high, because the frequent intersections and the orthogonal geometry of the grid pattern help pedestrians with detecting orientation and selecting the path to desired destinations [44]. This is developed by decomposing a metropolitan area into several city blocks, and a city block is further decomposed to multiple building blocks to create the hierarchy of the city as shown in Fig. 1b. This pattern also serves the entire urban area evenly so that it retains lower traffic congestion than a centralized structure [15].

### Hierarchical orthogonal layout

**Fig. 8:**
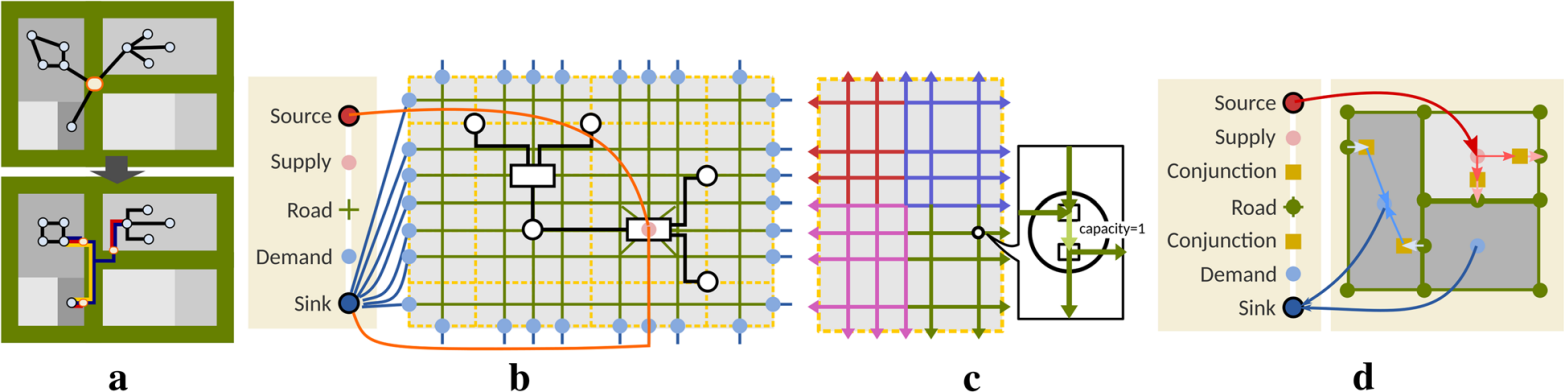
Examples of global and local edge routing, based on **a** a TreeMap decomposition. **b** A local flow network for guiding lines connecting reactions and chemical components settled on the block, and **c** restricted capacity to avoid multiple flows passing on one intermediates node. **d** A global flow network to build up line-set visualization for chemical components

To achieve the visual hierarchy, we decompose each urban block placed previously into several building (sub-)blocks using a TreeMap [36] to discriminate unique patterns in each categories. We then obtain a grid-like road network for edge routing based on this decomposition. Within each building block, we apply a compact orthogonal graph layout algorithm [55, 57] to each individual pathway sub-network to create well readable sub-drawings. The gray rectangles of the top image in Fig. 8a show an example of decomposition by TreeMap. Suppose that the orange node here is an important metabolite connected to reactions in multiple categories and the blue nodes indicate either a unique or an unimportant metabolite in *V*_*D*_. To reduce the visual complexity, we place this orange node on the boundary of each block to emphasize its difference (see the bottom image in Fig. 8a), while still showing the connectivity using the edge decomposition scheme described in “Overview of Metabopolis” section. The process to route edges consists of two steps, including building local flow networks *G*_*M*_=(*V*_*M*_,*E*_*M*_) and global flow networks *G*_*N*_=(*V*_*N*_,*E*_*N*_), respectively. We first build a local flow network *G*_*M*_ (“Local flow network for lane generation” section) of each building block to find optimal flows leading to metabolites on the block boundary (conjunction). Each flow thus corresponds to one directed edge connected to the metabolite as the red edges shown in Fig. 8a). Based on these edges, we then construct global flow networks *G*_*N*_ (“Global flow network for edge routing section) to distribute the flows used to connect chemical components appearing in different categories (see blue and yellow edges in Fig. 8a).

### Local flow network for lane generation

Once we have computed the orthogonal layout in each block, we are ready to arrange the nodes on the border and the edges connecting them. Our idea here is to formulate this problem as a local flow network and find its optimal flow paths that guide the shapes of directed edges by minimizing the intersections between the drawn orthogonal layout. The problem is solved by seeking minimum cost maximum flow using the successive shortest path algorithm [64]. Figure 8b shows an example of our local flow network, containing a source (red circle), a sink (blue circle), and several supply (pink circle), demand (aqua circle), and road (green diamond) nodes *v*_*m*_ in *V*_*M*_. The source and sink nodes are mandatory for the flow network algorithm, while we assign supply nodes to those reaction nodes connected to the metabolites on the block boundary and demand nodes to those possible candidates normally distributed on the boundary.

Since our goal here is to create a grid network suitable for winding an edge to avoid intersections with the drawn orthogonal layout, we build the network *G*_*M*_ by sampling intermediate horizontal and vertical lines between two consecutive nodes along *x* and *y* axes. The direction of each edge *e*_*m*_∈*E*_*M*_ is further decomposed into four as shown in Fig. 8c to avoid unexpected long edges across the block. Moreover, each intermediate sample is also decomposed by adding an additional edge with capacity one, to avoid multiple passing flows. We complete the network construction by adding directed edges from the source to supply nodes, supply nodes to their adjacent road nodes, and demand nodes to the sink node. Note that the orange line in Fig. 8b represents a flow example from the source to the sink. Our final task here is to assign appropriate capacities and weights to each edge *e*_*m*_ in the network. To ensure every supply will exactly lead to one demand on the boundary, we assign a capacity of one to each edge *e*_*m*_ to avoid multiple flow paths. Meanwhile, we define the corresponding weight function *w*(*e*_*m*_) for each edge *e*_*m*_=(*v*_*a*_,*v*_*b*_) as:

10 $$\begin{array}{@{}rcl@{}} \scriptsize  \begin{array}{ll} p |v_{a}-v_{b}| + q {\sum_{h \in H} c_{h}(e_{m})\cdot\bigl(|\frac{\vec{e_{m}}\cdot\vec{l_{h}}}{|\vec{e_{m}}||\vec{l_{h}}|}|+1\bigr)}, & \text{if \(e_{m} \in E_{M}\)}, \end{array}  \end{array} $$

to penalize if the edge is long or has intersections with existing orthogonal layout. Note that *c*_*h*_(*e*) counts to one if edge *e*_*m*_ is intersected with any edge *l*_*h*_ in the orthogonal layout, while we assign smaller penalty if the crossing angle is close to 90^∘^.

## Inter-block network layout

Until here, the basic information of node relationship is presented (black arrows in the result images). To highlight the duplicated metabolites, we incorporate additional colored edges between duplicated metabolites in different urban blocks in order to present the transaction flows between a pair of reactions sharing the same metabolites.

### Global flow network for edge routing

Again, we construct global flow networks for connecting pairs of identical chemical components appearing in different urban blocks (see blue and yellow edges in Fig. 8a). This is achieved by individually building a flow network from one block to the remaining blocks to visualize the global connectivity from one category to the others. The primary idea of this design is to control the edge density on a single road, to avoid accumulated flows by specifying a maximum flow capacity on each edge.

Figure 8d shows how our global flow networks are created. In contrast to the local network, the global one contains one more node component, the conjunction nodes (yellow square), whose coordinates are computed from the local network in “Local flow network for lane generation” section. We construct a different grid network by plotting all conjunction nodes and corner nodes of building blocks, to build the grid network by connecting nodes along the building block boundaries (see the green network in Fig. 8d). We then add bidirectional edges to our network by referring to the connectivity of this green mesh. The global network is finalized by adding directed edges from the source to supply nodes, supply nodes to the corresponding conjunction nodes, conjunction nodes to their demand nodes, and finally the demand nodes to the sink node.

To ensure that every supply has sufficient capacity to connect to other blocks, we compute the capacity *cap* of each supply edge by summing the connectivity from the source block to the other blocks, while we assign the corresponding exact value to the demand edges. Other edge capacities are assigned as:

11 $$\begin{array}{@{}rcl@{}} \scriptsize  \text{cap}_{g}(e_{n}) = \left\{ \begin{array}{ll} \text{capacity\_from\_reaction}(e_{n}), & \text{if \(e_{n} \in S\)}\\ \text{capacity\_to\_reaction}(e_{n}), & \text{if \(e_{n} \in D\)}\\ \text{MAX\_CAPACITY}-u(e_{n}), & \text{if \(e_{n} \in B\)}\\ \infty & \text{otherwise}, \end{array} \right.  \end{array} $$

where *u*(*e*) indicates the value of used capacity from the computed networks, and *S*, *D*, and *B* are edges connected to the supply, demand, and road nodes, respectively. The edge weight function *w*_*g*_(*e*_*n*_(*v*_*a*_,*v*_*b*_)) is simply defined as |*v*_*a*_−*v*_*b*_|. The MAX_CAPACITY is a user-defined constant to limit the maximum flow on a road. The problem is again solved by the same classic flow algorithm [64].

## Implementation and enhancement

Our system has been implemented on a desktop PC with an AMD Ryzen 7 1800X CPU (8 Cores, 16 Threads) and 64GB RAM. The source code was written in C++ using Qt Graphics for rendering maps and user interface, Boost Graph Library for the graph data structure and minimum cost maximum flow algorithm, CGAL for computational geometry algorithms, and IBM CPLEX for the MIP-based optimization. Since our framework is designed to incorporate different graph layout algorithms for decomposed sub-graphs, we introduced orthogonal layout algorithms here due to their high computational complexity and familiarity in most of the database [3, 4]. Users are allowed to choose preferable compact orthogonal layout by selecting the open-source HOLA package [55] or the commercial library yFiles [57] in the system, but only HOLA is chosen for the visualization in this paper.

### Supported file format

The biological relationship is often recorded using the *Systems Biology Markup Language* (SBML) [65], an extended XML file format, which we also use as the input data format of our system. To further increase the popularity of the proposed technique, we store our layout using an extended *Systems Biology Graphical Notation Markup Languages* (SBGNML) [66], which is a standard visual representation of SBML. Our system is open-source and has been put on a GitHub repository named *Metabopolis* [67].

### Setting of the MIP optimization

Since the space partitioning scheme in Metabopolis is achieved by solving a MIP problem, we cannot only control solution space, but profit from algorithmic speedup by solvers such as IBM CPLEX, Gurobi, SCIP, or Xpress. As compared to other constrained optimization techniques such as greedy algorithm, simulated annealing approach, or genetic algorithm, MIP is more prominent here in the sense that it also provides the optimal solution by time, which cannot be achieved or predicted by other heuristic approaches. Especially in one of our use cases, where we generate high quality maps to serve as an overview map for databases, we need a reliable approach that generates a diagram with high readability. With modern MIP techniques, the formulation can be also relaxed to find a feasible, but possibly suboptimal, solution in shorter time. Moreover, it turned out that *overlap-free block placement (CH2)* constraints are bottlenecks in our approach. We can formulate them as lazy constraints in modern MIP solvers so they will be only included when the result violates the constraint. This is done together with introducing *pairwise relative positioning (CH3)*, to restrict the solution space in a reasonable way.

Our constrained floor-planning algorithm relies on an input graph skeleton for guiding block placement, which helps preserving the mental map of users as well as limiting the search space for the NP-hard floor-planning problem. If all edges of the graph are removed, the problem will then become conventional floor-planning problem, which may not be sufficiently solved using MIP solvers. We suggest users to add at least one edge to each block for better control of the layout.

### Content-adaptive navigation

We also incorporate a content-adaptive navigation scheme to support the layout navigation. The zoom-levels have been categorized into three, including the overview level, the intermediate level, and the detailed level, where the font size and intensity are changed accordingly. Four color schemes, including the predefined, the monotone, the pastel, and the *ColorBrewer* color sets, are incorporated for selection. The high level category names (ex. names of ontology labels or compartments) are placed at the center of each block, while we rotate the name 90 degrees when the block is vertically extremely long. At the intermediate level, we also enlarge the reaction names by a factor of 3, so that names become readable when the screen space is sufficiently large. The font intensity of category names is decreased as the zoom level increase, while the names of metabolites and reactions change contrarily. Metabopolis also includes traditional interactions such as highlighting reactions and metabolites, as well as corresponding neighbors.

## Experimental results, evaluation, and discussion

The Metabopolis diagram representing the major categories of the human metabolic network is shown in Fig. 2. The red route in *Glycolysis Gluconeogenesis* (orange block) shows the cytoplasmatic oxidation of glucose to produce ATP. Using our diagram (each category is represented by a different color), we immediately observe that the entire process only happens in *Glycolysis Gluconeogenesis* since all positions of the corresponding reactions are constrained within one block. However, the produced ATP is placed on the boundary of the block here, which means that it also serves as reactant of other reactions outside the *Glycolysis Gluconeogenesis*. In biology, ATP is the key energy molecule utilized to drive other biological reactions. The green route shows an example of ATP conversion in the cytoplasm to enable urea synthesis (the blue route). Compared to *Glycolysis Gluconeogenesis*, reactions involved in urea synthesis are more complicated, since they require to bring ammonia from mitochondria to the cytoplasm to complete the synthesis. This is visualized by showing the reactions involved actually belong to *Urea Cycle*, *Alanine and Aspartate Metabolism*, and *Transport* pathways in our diagram.

**Fig. 9:**
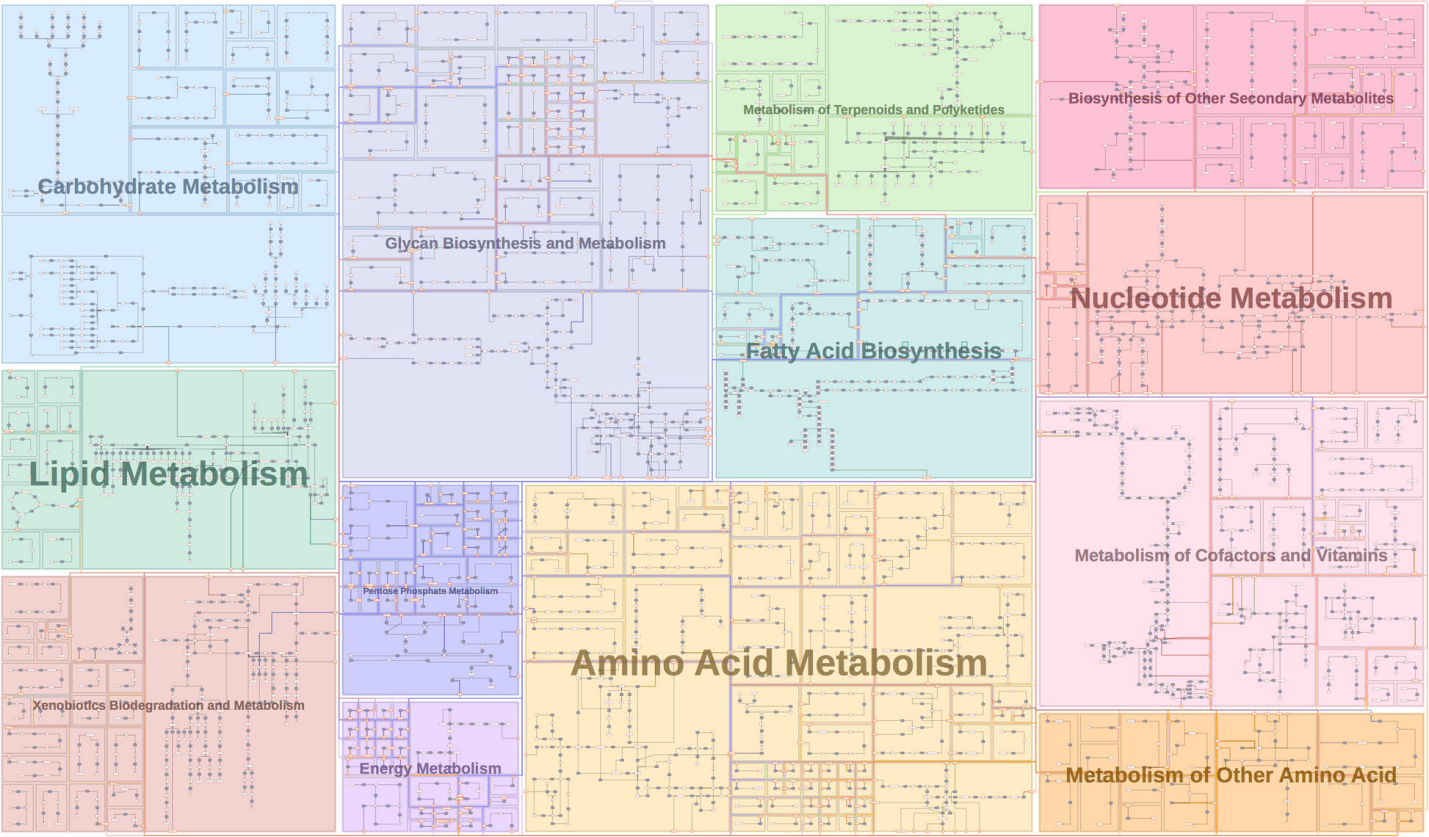
Redrawing the human metabolic pathway map of KEGG [68] by using Metabopolis. Blocks with warm colors are grouped to the right and cool colors are grouped to the left as originally designed in the KEGG overview map

**Fig. 10:**
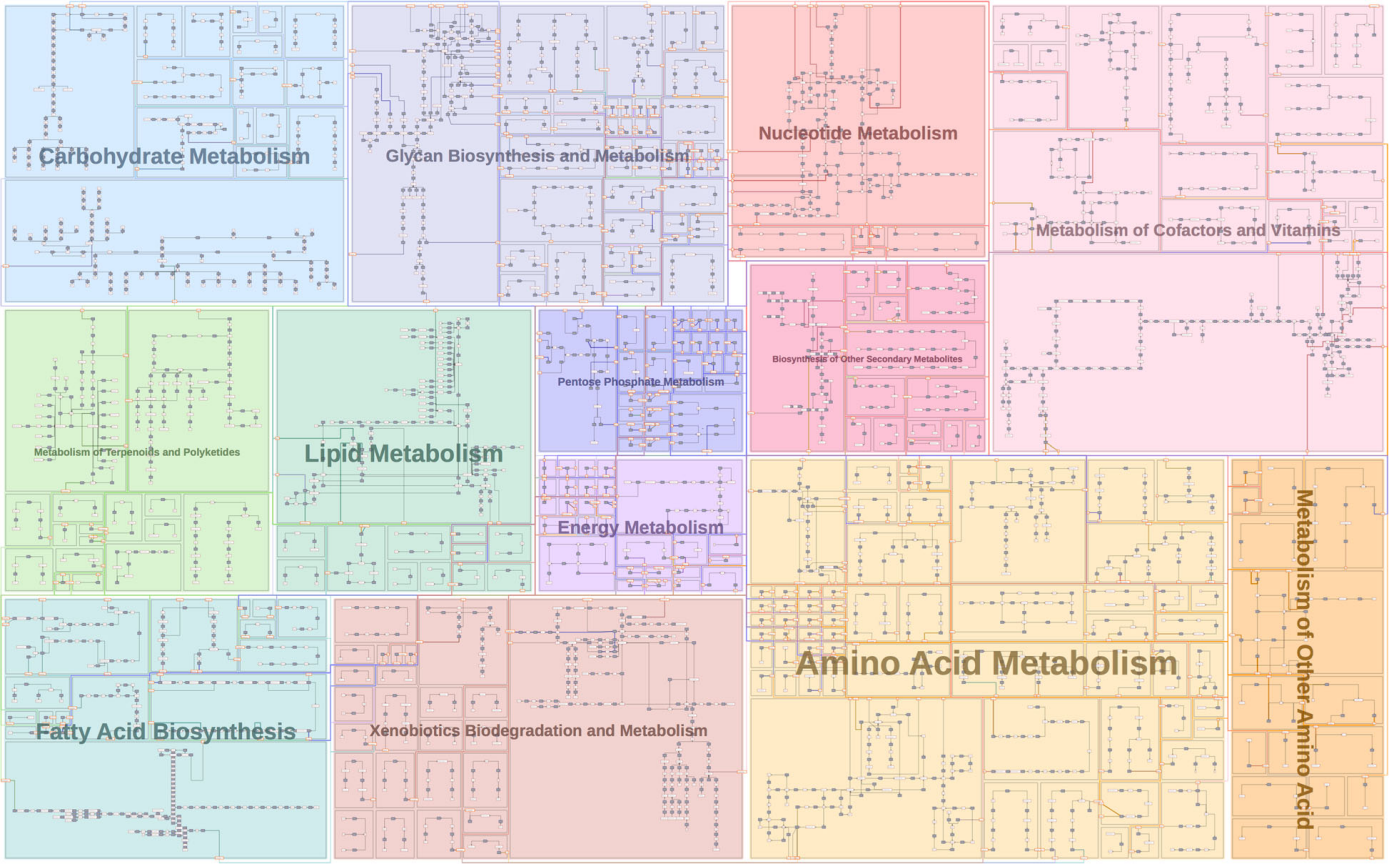
Compared to Fig. 9, the relative positions of urban blocks are manually adjusted by referring to the semantic categories defined in the KEGG overview pathway map [68]

Figure 9 is an example of reproducing the KEGG overview pathway map of human metabolic pathways, and the diagram is fully computed using Metabopolis without manual adjustment. Metabopolis allows us to automatically duplicate the nodes by setting an experimental threshold 80 to the frequency of inter-category edges. This result is similar to the original *KEGG* map [68], since blocks with warm and cool colors are grouped together as originally designed. In addition to the automatic layout computation, Metabopolis also supports several user interactions. This allows users to assign strongly connected blocks as neighboring blocks, highlight connected reactions or metabolites, and relationships between reactions and metabolites. Figure 10 shows the result, where we parsed the graph data and assigned the block position by referring to the category position from the metabolic pathway diagram of KEGG [68]. We also borrow the color coding from KEGG and assign the initial block center by calculating the average of nodes involved in the same semantic categories.

**Fig. 11:**
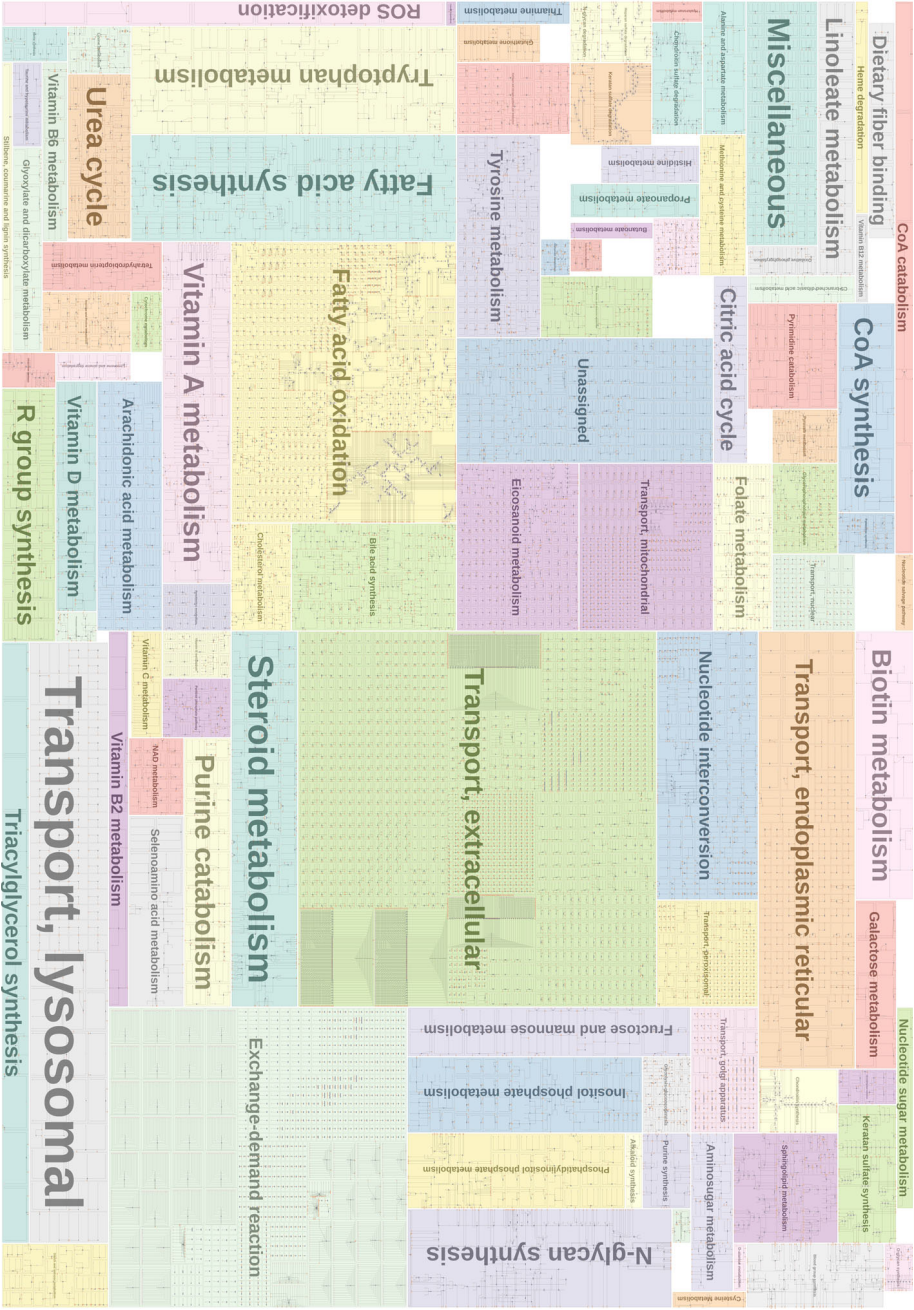
Human metabolism reconstructed from the Recon project [4, 5]

Figure 11 shows the same human metabolic network *ReconMap* [4, 69]. To the best of our knowledge, this is the first automatically generated pathway diagram that shows the entire human metabolic network in a clear, orthogonal, and hierarchically structured fashion.

Since pathway diagrams provide a way to understand fundamental metabolisms and the responses of diseases to drugs, researchers increasingly pursue network-centric approaches to investigate the functionality and controllability of pathways. There are two typical pathway categories: small pathway maps (e.g., citric acid cycle) often summarize a set of core chemical reactions and large pathway maps (e.g., human metabolic pathways), which collect extensive processes among one species. The former pathways such as Fig. 2 are often used to describe a specific biochemical theme and the latter such as Figs. 9 and 11 are commonly referred to imagine how the functions of a metabolite would influence the entire metabolism. Attributes of chemical components and reactions, such as cell compartments (organelles) and *biological pathway ontology* (the standard identifiers), allow researchers to share the corresponding knowledge by querying for categories of interest [70].

Table 1 summarizes the size of the metabolic networks visualized in this paper, where |*V*|,|*E*|, and density (*Den*) correspond to node number, edge number, and density before node duplication, respectively. Similarly, |*V*_*D*_|,|*E*_*D*_|, and density (*Den*_*D*_) are the respective values after node duplication. The edge density function for *Den* and *Den*_*D*_ is defined as |*E*|/(|*V*|^2^−|*V*|) [33] in Table 1. Note that here we decompose a hyperedge of a reaction into multiple edge segments used to connect metabolites to construct our network. The duplication allows us to reduce graph density to untangle edge crossing in the visualization.

**Table 1:** The number of nodes (|*V*|), edges (|*E*|), and density (*Den*) before and after node duplication, while |*C*| shows the number of categories of our sample images

	Before duplication/After duplication			
	|*V*|/|*V*_*D*_|	|*E*|/|*E*_*D*_|	*Den*/*Den*_*D*_	|*C*|
Figure 2	593/948	1244/1635	0.354%/0.182%	11
Figures 9-10	3679/5954	4008/4010	0.030%/0.011%	13
Figure 11	12503/22860	31540/42007	0.020%/0.008%	100

Table 2 presents the computation times of the figures shown in this paper. Although it still takes a few hours to compute the entire human metabolic network, our approach provides the first computational tool to generate pathway maps of that size automatically rather than in months of tedious manual work. In addition, we also visually compare the results generated using *Prefuse Force-Directed Layout* [25], *Compound Spring Embedder Layout* (CoSE) [71], and Metabopolis in Additional file 1: Appendix A.

**Table 2:** Computation times of each algorithmic step of the sample images (in seconds)

	floorplan (“Constrained floor-plan problem” section)	adjustment (“Fine block adjustment” section)	orthogonal (“Hierarchical orthogonal layout” section)	local flow (“Local flow network for lane generation” section)	global flow (“Global flow network for edge routing” section)
Figure 2	0.94	0.62	0.06	0.03	5.54
Figure 10	10.65	0.62	33.17	0.75	60.01
Figure 9	11.14	0.88	33.08	0.78	61.15
Figure 11	5630	643	193.15	191.44	1634

For validating our approach we reached out to domain experts who manually create pathways in their daily research and interviewed them after they investigated several maps generated using Metabopolis. This includes one of our co-authors, Dr. Filipa L. Sousa, one member from ReconMap [4], three members from KEGG [3], and one member from CellDesigner [22].

Dr. Sousa mentioned that compared to conventional network visualization such as the overview map from *KEGG pathway maps* [3] and a force-directed layout from *Cytoscape* [25]), one has to get used to the layout of Metabopolis initially. Then, it becomes easier to adapt to the proposed layout and visualization rules since it restricts the positions of categories. It is also an advantage to select categories and arrange adjacent categories to personalize the pathway maps. Regarding the design principles, grouping of pathways within the same category is a good option to emphasize chemical components in the same categories. For a larger map, since each reaction is represented in its full form, node duplication is helpful while some maps might become too confusing. An automatic scheme by cloning commonly duplicated metabolites from the manually-created maps is good so that it is not infeasible both from the biological and from the layout side. One may need to learn the decomposition scheme of long directed edges, while it is understandable through experience. The background line-set feature, which uses line width to represent the number of reactions a metabolite is involved in, provides a good visual guidance to which metabolite has the strongest connectivity to other metabolites when visualizing a certain compartment/category of reactions in detail. Nonetheless, if someone is not familiar with the nomenclature commonly used in metabolic networks, visually searching reactants and products of a reaction on a pathway map is impractical compared to name query. However, once the right metabolite is chosen, it becomes intuitive to read the connectivity and the belonging category through Metabopolis. Color-coded bundled edges is not intuitive if the map is large, while together with color highlighting it is clearer.

Dr. Ronan Fleming from *ReconMap* [4] has initiated the project for manually drawing human metabolic pathways in a full form to support comprehensive understanding of the network content together with omics data and simulation results. He expressed his great interest to Metabopolis since he did not receive more than one feedback from the *Graph Drawing Contest 2017* [14] and considered our technique the first automatic approach targeting this large network. He confirmed that this first work opens new opportunities in the bioinformatics community. Since Dr. Fleming’s team has used *CellDesigner* [22] for manually generating pathway layout, we also interviewed Dr. Akira Funahashi, the corresponding author of *CellDesigner*. Dr. Funahashi is impressed by the layout controllability of the maps generated by Metabopolis, while he suggests to fully control the sub-patterns to make the maps easier to memorize and relax the shapes of rectangles to create distinct block structures. Dr. Minoru Kanehisa and his team members have been working on manually creating pathway maps for *KEGG pathway database* [3] for 20 years because they believe the quality of hand-crafted pathway maps is better. With our technique, they see the potential to automatically arrange reactions in the same organelle closer to each other and provide readers with an opportunity to learn pathways in a top-down fashion.

Although all domain experts agree that the manually adjusted layout still performs better in terms of visual quality (e.g., preserving citric acid cycle as a circle in the drawing), they have inspected our automatically generated diagrams with great interest and forsee its future potential. We were recommended to include the layout algorithm in conventional software packages, so that both biologists and bioinformatics community can connect our layout to existing biological databases, and to incorporate search functions to facilitate sophisticated pathway analysis, including retrieving additional information regarding genes, catalyzing reactions and other biological information such as KOs, COGs, etc. Advanced editing is also advised to revise the datasets. After the discussion, we have improved the display of all nine combinations of directional/bidirectional edges shown in Fig. 4b, by generating one line between pairs of chemical components and thus producing a dense line drawing when the diagram is large. We then bundle these lines to a certain width, while allowing users to highlight and extend their target of interests through our user interface, or click on the chemical component to see how it is distributed as the conventional hand-drawn pathway maps provided.

## Conclusion and future work

This paper presents the very first approach to automatically design biological pathway diagrams in urban map style by integrating a constrained floor-plan technique together with a visual hierarchical representation using a network flow algorithm for edge routing. Our approach ensures the appropriate partitioned space for categories with biological meanings, while seeking the balance between size of categories and neighborhood relationship. This has been done by formulating the problem as a mixed integer programming model. We devise a visual hierarchical design for metabolites by borrowing the concept of urban maps, where we reasonably bind the line sets connecting to identical metabolites to restricted roads bounded by categories. The paths have been distributed by flow-network algorithms. An interface for editing, navigating and highlighting target metabolites is also provided for further customization.

Metabopolis is the first work aiming at automatic generation of entire large metabolic pathways, thus we open a myriad of opportunities for a domain where there were only few and mostly non-customized manually designed maps until now. Future work will involve the creation of a visual web repository, where biologists can continuously add or create their maps together with sharing the maps with the rest of the community. Everyone is welcomed to contribute to the knowledge collection and suggest preferable visual representations since the layout is expected to be created associated with preferred tasks. We will allow users to create and update the diagram with domain-specific information so that we can automatically transform this knowledge to mathematical equations for the layout optimization. To achieve this, we have been in collaboration with CellDesigner [22] on plugin development, and plan to release packages for other popular tools such as *Cytoscape* [25] to initiate the dynamic pathway layout framework in the biology community. We have released our initial system on GitHub [67], a tutorial (Additional file 1: Appendix B), and will complete the entire framework by coupling with a version control system for pathway diagrams. Another goal is to reach out to the structural biology community by integrating the pathway diagrams with structural data of the chemical components. By extending the rectangular blocks, we target more complex geometry for representing the context of the chemical components such as cell compartments, cell types, or organs.

## Additional file

Additional file 1: Appendices In Appendices, we first compare and describe the experimental results generated using conventional layout algorithms and our approach. Afterward, a tutorial of Metabopolis has been included to explain the usage of the software. (PDF 7840 KB)

## Abbreviations

ATP: Adenosine triphosphate
CPLEX: IBM ILOG CPLEX optimization studio
Gurobi: The gurobi optimizer
HOLA: Human-like orthogonal network layout
MIP: Mixed-integer programming
SBGN: Systems biology graphical notation
SBGNML: Systems biology graphical notation markup languages
SBML: Systems biology markup language
SCIP: SCIP optimization suite
VLSI: Very large scale integration
XML: Extensible markup language
Xpress: FICO Xpress optimization
