# Supplementary material for: Metabopolis: scalable network layout for biological pathway diagrams in urban map style
Source: BMC Bioinformatics. 2019 Apr 15;20:187. doi: 10.1186/s12859-019-2779-4 (PMC6466808; doi:10.1186/s12859-019-2779-4)
Supplement: Supplementary file 1 — In Appendices, we first compare and describe the experimental results generated using conventional layout algorithms and our approach. Afterward, a tutorial of Metabopolis has been included to explain the usage of the software. (PDF 7840 KB) [file 12859_2019_2779_MOESM1_ESM.pdf]

Appendices

Appendix A: Visual Comparison with Conventional Layout Algorithm

We also compare the maps generated by Metabopolis with several conventional layout algorithms, including *Prefuse Force-Directed Layout* [25], *Compound Spring Embedder Layout* (CoSE) [70], and *Orthogonal Layout* [57] and applied the algorithm on the KEGG dataset [68] and the *ReconMap* dataset [4]. Our algorithm, as a pioneering automatic approach, aims to visualize large metabolic pathway such as human metabolic pathways. Although it did not produce the same or better quality as hand-drawn maps, it does provide an underlying sketch for refining the layout. This will drastically reduce the working time for creating a hand-drawn map. Table 3 presents the computational times of each listed conventional layout algorithm and the maps generated by Metabopolis. Metabopolis is not the fastest algorithm among the four algorithms, yet its result has better space coverage together with a clear underlying category information. For *Compound Spring Embedder Layout* and *Orthogonal Layout*, no feasible results were computed within 5 hours.

Table 3 Computation times of conventional algorithms and Metabopolis (in seconds). The datasets include the pathways in Fig. 3, KEGG overview map (KEGG) [68] , and Human Metabolic Pathways (HMP) [4].

	Forced-directed	CoSE	Orthogonal	Metabopolis
Fig. 3	< 1 (Fig. 15)	< 1 (Fig. 15)	< 1 (Fig. 15)	2 (Fig. 3)
KEGG	1 (Fig. 12)	3 (Fig. 16)	216 (Fig. 13)	107 (Fig. 9)
HMP	70 (Fig. 14)	over 5 hrs	over 5 hrs	8291 (Fig. 11)

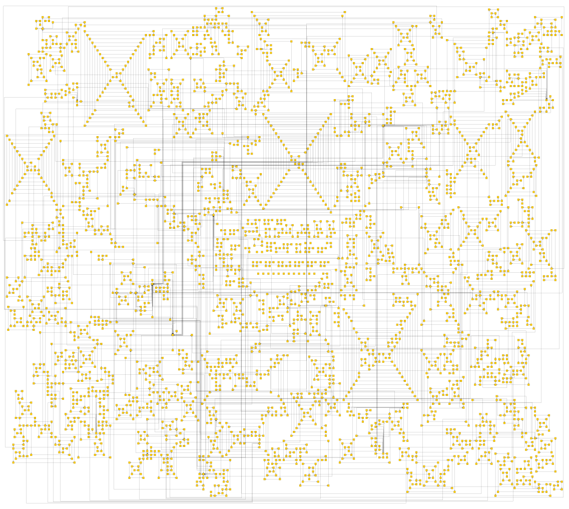

Figure 13 *Compact Orthogonal Layout* [57] of *KEGG* dataset [68].

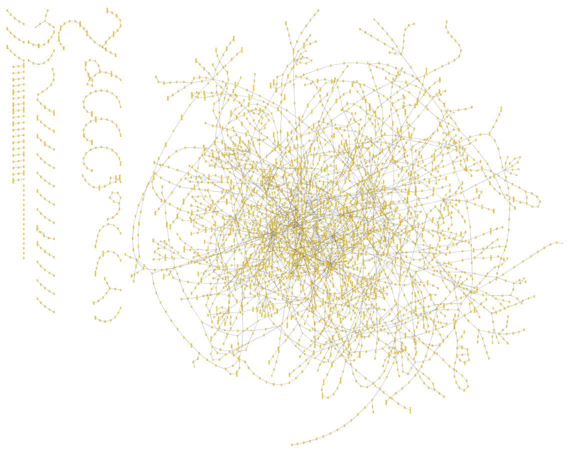

Figure 12 *Prefuse Force-Directed Layout* [25] of *KEGG* dataset [68].

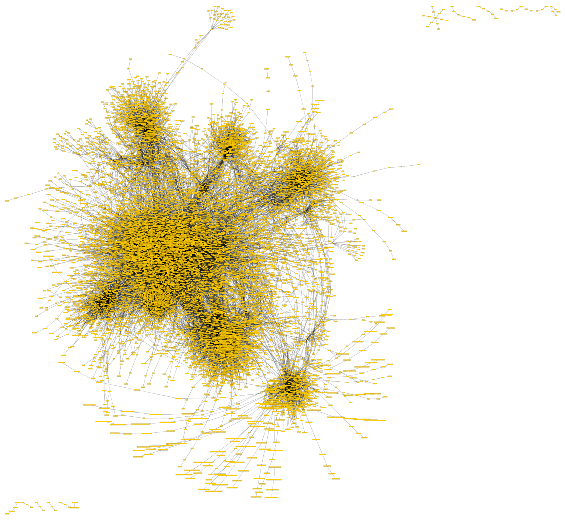

Figure 14 *Prefuse Force-Directed Layout* [25] of *ReconMap* dataset [4].

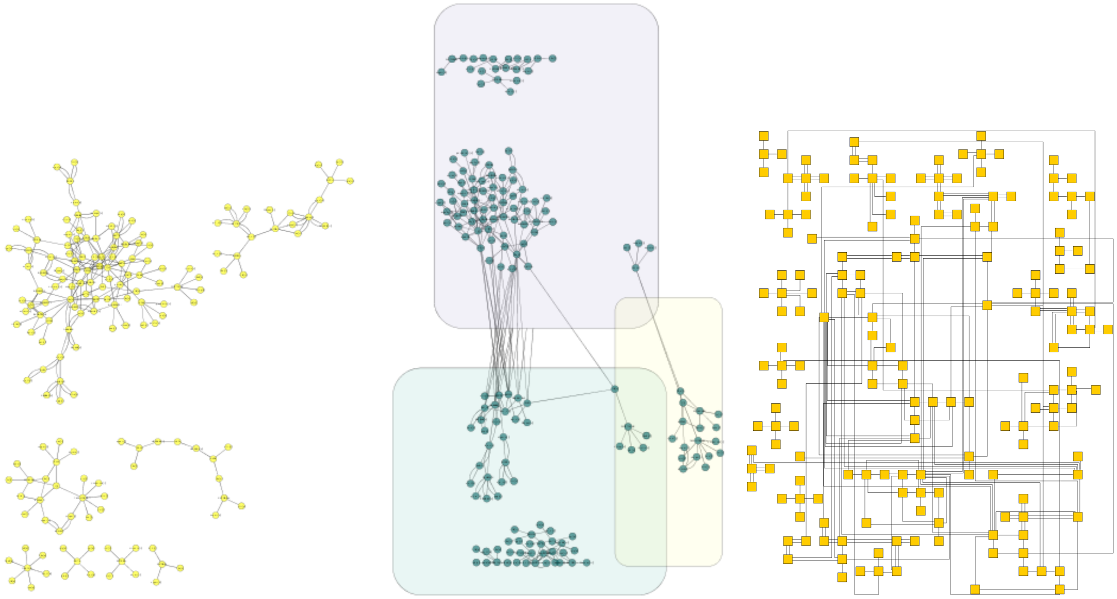

(a) (b) (c)

Figure 15 Additional layout of the dataset in Fig. 3, including (a) *Prefuse Force-Directed Layout* [25], (b) *Compound Spring Embedder Layout* (CoSE) [70], and (c) *Compact Orthogonal Layout* [57].

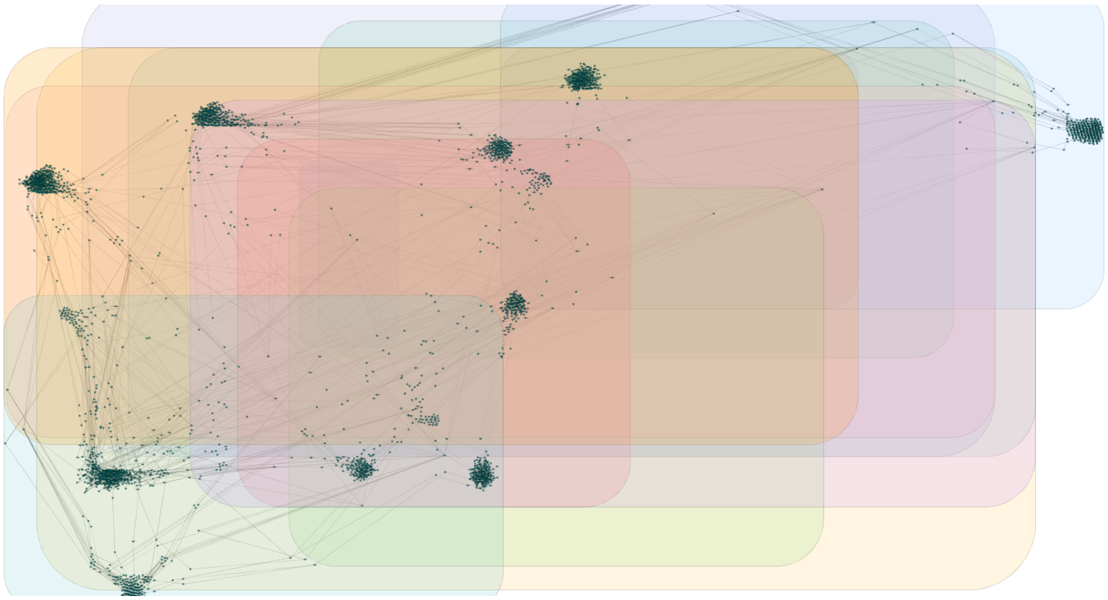

Figure 16 *Compound Spring Embedder Layout* (CoSE) [70] of *KEGG* dataset [68].

Appendix B: A tutorial of Metabopolis

After Meabopolis is alunched, the left window shows the current pathway diagram and the right window is the control panel of the system.

Step 1: Browse a folder to load a set of interesting data. Alternatively, the degree of duplication can be specified by uploading a list of metabolite types or by using a user-specified value. Then the data can be loaded by pressing the *Load* button (see Figure 17).

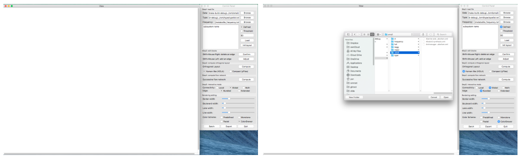

(a) (b)

Figure 17 (a) User interface of Metabopolis. (b) Load sets of pathway files from a folder.

Step 2: Edit the relationship graph from the automatically computed one and compute the balanced space for the orthogonal layout. To delete an edge, users need to press *Shift Key + Right Mouse Button* on an *Edge*. To add an edge, users need to press *Shift Key + Left Mouse Button* on a *Node*. Then press the *Confirm* button to compute graph layout and the *Adjust* button to adjust the box size.

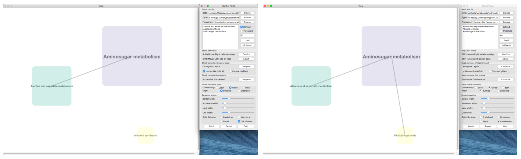

(a) (b)

Figure 18 (a) Initialize block position after the skeleton is generated. (b) Add or deleted edges of the skeleton.

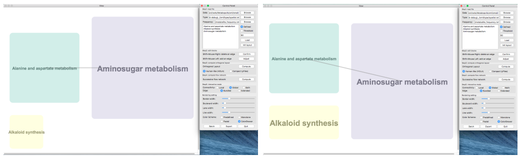

(a) (b)

Figure 19 (a) Compute the block position by referring the connectivity of the skeleton. (b) Adjust block size to fulfill the balanced rectangular space.

Step 3: Compute the orthogonal layout by first selecting the type of the layout. Currently, only *HOLA* and *yFiles Compact Orthogonal Algorithm* are supported.

Step 4: Compute the edge routing of important metabolites associated with different categories. Here users are allowed to adjust the border, boulevard, lane, and line width to advance the preferred frame of the layout.

Step 5: Finally, in the interaction mode, users can select *Local*, *Global*, or *Both*, to highlight the connection of metabolites in one category, in multiples categories, or both. Users are allowed to bundle the edges to see the overview or extend the bundled edges to see more detailed edge connectivity (see Figure 22).

Note that the layout can be automatically computed by clicking on the left-bottom button *Batch*. Press *Export* to export svg or png images, which will be stored at $METABOPOLIS/svg/$

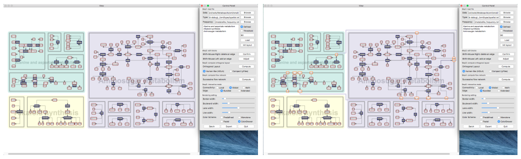

(a) (b)

Figure 20 (a) Apply orthogonal layout on each subgraph. (b) Route edges to connect metabolites belonging to different categories.

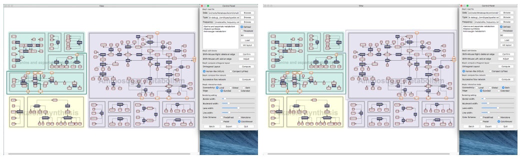

(a) (b)

Figure 21 (a) Show local edge routing (b) Show global edge routing.

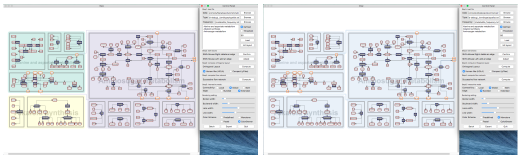

(a) (b)

Figure 22 (a) Extend the local edges go see each single edge connection (b) Apply different color scheme.

Figure 23 shows a pathway diagram, including subsystems *Alanine and aspartate metabolism* (green), *Alkaloid synthesis* (yellow), and *Aminosugar metabolism* (purple). Orange vertices, such as $atp[c]$, $ac[c]$, $gln_L[c]$, and $glu_L[c]$, which are aligned on the block boundary are metabolites involved between green and purple blocks. The purple line connecting $ac[c]$ produced from the AGDC reaction goes to reaction $RE2642$ in the green block. This shows that the reactant $ac[c]$ of $RE2642$ could come from the AGDC reaction. With our design, users do not need to trace a long edge to find the metabolite name, but only need to check the neighborhood. Our interface also provides users to highlight the connecting reactions and metabolite for investigation.

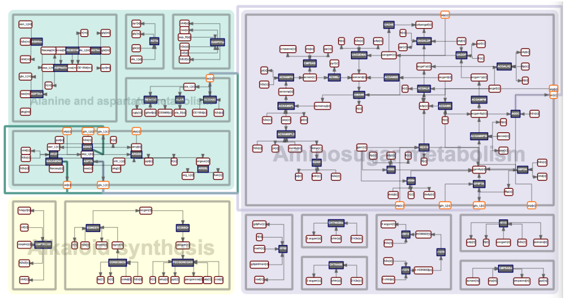

Figure 23 An simple example generated by Metabopolis.
